# Lung EC-SOD Overexpression Prevents Hypoxia-Induced Platelet Activation and Lung Platelet Accumulation

**DOI:** 10.3390/antiox13080975

**Published:** 2024-08-10

**Authors:** Daniel Colon Hidalgo, Mariah Jordan, Janelle N. Posey, Samuel D. Burciaga, Thi-Tina N. Nguyen, Christina Sul, Caitlin V. Lewis, Cassidy Delaney, Eva S. Nozik

**Affiliations:** 1 Department of Medicine, Division of Pulmonary and Critical Care, University of Colorado Anschutz Medical Campus, Aurora, CO 80045, USA; daniel.colonhidalgo@cuanschutz.edu; 2 Cardiovascular Pulmonary Research Group, Departments of Pediatrics and Medicine, University of Colorado Anschutz Medical Campus, Aurora, CO 80045, USA; 3 Department of Pediatrics, Division of Neonatology, University of Colorado Anschutz Medical Campus, Aurora, CO 80045, USA; 4 Department of Pediatrics, Division of Critical Care, University of Colorado Anschutz Medical Campus, Aurora, CO 80045, USA

**Keywords:** platelets, pulmonary hypertension, superoxide dismutase, hypoxia

## Abstract

Pulmonary hypertension (PH) is a progressive disease marked by pulmonary vascular remodeling and right ventricular failure. Inflammation and oxidative stress are critical in PH pathogenesis, with early pulmonary vascular inflammation preceding vascular remodeling. Extracellular superoxide dismutase (EC-SOD), a key vascular antioxidant enzyme, mitigates oxidative stress and protects against inflammation and fibrosis in diverse lung and vascular disease models. This study utilizes a murine hypobaric hypoxia model to investigate the role of lung EC-SOD on hypoxia-induced platelet activation and platelet lung accumulation, a critical factor in PH-related inflammation. We found that lung EC-SOD overexpression blocked hypoxia-induced platelet activation and platelet accumulation in the lung. Though lung EC-SOD overexpression increased lung EC-SOD content, it did not impact plasma extracellular SOD activity. However, ex vivo, exogenous extracellular SOD treatment specifically blunted convulxin-induced platelet activation but did not blunt platelet activation with thrombin or ADP. Our data identify platelets as a novel target of EC-SOD in response to hypoxia, providing a foundation to advance the understanding of dysregulated redox signaling and platelet activation in PH and other chronic hypoxic lung diseases.

## 1. Introduction

Pulmonary hypertension (PH) is an incurable condition characterized by progressive remodeling of the pulmonary vascular bed, culminating in right ventricular failure in the most severe cases [1,2,3]. Available treatments do not adequately improve outcomes, supporting the need to better understand the molecular mechanisms driving the disease process [3,4,5]. Accumulating evidence supports the critical role of both systemic and pulmonary vascular inflammation in the pathogenesis of PH [6,7,8]. Increased circulating proinflammatory chemokines and cytokines and the accumulation of lung immune cells are well described in patients with PH [9,10]. In preclinical models, systemic and pulmonary vascular inflammation precedes pulmonary vascular remodeling and PH, suggesting that inflammation is a driver rather than a consequence of the disease [10,11]. 

Inflammation and oxidative stress are intimately linked. Inflammation results, in part, from increased platelet and leukocyte activation, which generates reactive oxygen species (ROS), influencing redox-sensitive signaling molecules that drive further inflammation [12,13]. Extracellular superoxide dismutase (EC-SOD) is a major regulator of lung and vascular oxidative stress, scavenging ROS by catalyzing the dismutation of superoxide to hydrogen peroxide and oxygen [14,15]. Vascular EC-SOD is produced predominantly in vascular smooth muscle cells and secreted into the extracellular compartment where it is bound to the matrix (ECM) via its positively charted heparin-binding domain (HBD). EC-SOD can also be released into the extracellular fluid following cleavage of the HBD [16,17]. EC-SOD protects against inflammation and fibrosis in a wide range of lung and vascular diseases [18,19,20]. We have previously shown that mice overexpressing lung EC-SOD are protected from chronic hypoxic PH [21]; however, the effects of EC-SOD on early inflammation, as a mechanism for this protection, have not been fully elucidated. 

Beyond their role in thrombosis and hemostasis, platelets also mediate inflammation and immunity [22,23,24,25]. Platelet activation leads to the activation of the integrin αIIBβ3, release of alpha-granule stored proinflammatory chemokines and cytokines, including P-selectin and platelet factor 4 (PF4), and formation of platelet-leukocyte aggregates (PLAs) [26,27,28]. We have demonstrated that platelets are increased in the lungs of patients with end-stage PH (Delaney 2021). Additionally, others have shown that platelets are activated in patients with PH, demonstrated by increased platelet-derived proteins and microparticles in circulation and the formation of platelet-leukocyte aggregates [29,30]. In the murine hypobaric hypoxia model of PH, early hypoxia (3 days) leads to platelet activation and accumulation of platelets in the lung [31]. We recently demonstrated that platelets regulate early hypoxia-induced pulmonary interstitial macrophage expansion, a critical process in inflammatory-mediated PH [31]. Multiple signaling molecules involved in platelet activation are under redox control [32,33,34], providing a strong rationale to consider how EC-SOD content could impact platelet activation in response to hypoxia. We hypothesized that elevated lung EC-SOD content, which is protective in chronic hypoxic PH [21], would prevent early hypoxia-induced platelet activation and accumulation of lung platelets. To test our hypothesis, we utilized heterozygous mice that overexpress human EC-SOD via the surfactant protein C promoter (SPC) [21]. We exposed them to normoxia or hypobaric hypoxia and measured platelet activation. We evaluated the direct effect of extracellular SOD on platelet activation using an ex vivo model of platelet activation with exogenous SOD treatment. 

## 2. Materials and Methods

Mouse model: 8–12-week-old male and female wildtype C57Bl/6 mice (WT) and mice overexpressing lung EC-SOD (Tg) [21] bred at Denver altitude were exposed to either normoxia (ambient air) or hypobaric hypoxia chambers for 3 days (395 Torr; to simulate 10% FiO_2_) [21,35,36,37]. We previously demonstrated that EC-SOD activity is increased, and the secreted protein generated by human transgene in type II alveolar cells via the SPC promoter is distributed throughout the lung in the Tg mice [21]. The time points were selected based on published studies that demonstrated a transient peak in platelet activation and lung platelet accumulation at 3 days [31]. Mice underwent anesthesia with 1–2% isoflurane followed by euthanasia, and blood was collected and processed as described below. Mouse lungs were flushed with 10 mL of PBS and saved in Allprotect Tissue Reagent (Qiagen, Hilden, Germany) or prepared for paraffin-embedding and immunohistochemistry; briefly, lungs were inflation-fixed at 20 cm H_2_O for 30 min with 4% paraformaldehyde followed by 4% paraformaldehyde for 24 h before transfer to 70% ethanol. The Institutional Animal Care and Use Committee (IACUC) at the University of Colorado Anschutz Medical Campus approved all studies.

Protein isolation: Lung homogenates were generated from 30 mg of whole lung tissue in lysis buffer containing T-PER (Thermo Fisher Scientific, Waltham, MA, USA) with protease inhibitor cocktail (Sigma-Aldrich) and phosphatase inhibitor cocktails 2 and 3 (Sigma-Aldrich, Burlington, VT, USA) using the Bead Ruptor12 (Omni International, Kennesaw, GA, USA), operated at high speed for 45 s and then cooled on ice (×3 cycles). Following homogenization, samples were incubated for 30 min on ice, followed by centrifugation at 10,000× *g* for 5 min. Lung homogenate protein concentration was determined utilizing the Pierce Rapid Gold BCA protein assay kit (Thermo Fisher Scientific, Waltham, MA, USA) and stored at −80 °C until used in assays.

Preparation of mouse blood, platelets, and plasma: In anesthetized mice, blood was collected via terminal closed-chest cardiac puncture from the right ventricle following established procedures [36,38]. Platelet-rich plasma (PRP) was isolated through centrifugation of heparin-anticoagulated whole blood at 100× *g* for 16 min. To prevent platelet pre-activation, PRP was supplemented with PGI2 (1 μg g/mL, Cayman Chemical, Ann Arbor, MI, USA) and apyrase (0.02 U/mL, Sigma-Aldrich, Burlington, VT, USA), then incubated at room temperature (RT) for 3 min before further centrifugation at 2000× *g* for 2 min to obtain platelet-poor plasma (PPP). Plasma samples were preserved at −80 °C until use. Complete blood counts were analyzed from heparin-anticoagulated blood using the Heska HT5 hematologic analyzer (Loveland, CO, USA).

Assessment of platelet activation: Whole blood anticoagulated 1:6 with acid citrate dextrose (ACD) was mixed with 1 mL of warm Tyrode’s Buffer, pH 7.3 [composed of NaCl 129 mM, KCl 2.9 mM, MgCl_2_ 1 mM, NaH_2_PO_4_ 0.34 mM, NaHCO_3_ 12 mM, and glucose 5 mM]. This mixture was supplemented with PGI2 (1 ug/mL) and apyrase (0.02 U/mL) to prevent platelet pre-activation and allowed to incubate for 3 min at room temperature (RT). Afterward, the diluted blood underwent centrifugation at 150× *g* for 5 min at RT. The resulting platelet-rich plasma (PRP) was carefully collected, transferred to a fresh tube, and supplemented again with PGI2 (1 μg/mL) and apyrase (0.02 U/mL) for 3 min at RT. PRP samples were then centrifuged at 450× *g* for 10 min. The supernatant was removed, and the platelet pellet was resuspended in 500 μL of warm Tyrode’s Buffer, maintaining a temperature of 37 °C until further dilution for assay. Flow cytometry analysis involved diluting washed platelets (1 × 10^6^ platelets) in 100 μL of warm Tyrode’s buffer containing 1 mM CaCl_2_. Platelets were then incubated for 10 min at RT in the dark, along with anti-mouse CD41-BV421 antibody (BioLegend, San Diego, CA, USA, Clone No. MWReg30; 1:50), P-selectin-APC (Thermofisher, Waltham, MA, USA, Clone No. Psel.KO2.3; 1:25), and activated integrin αIIBβ3-PE antibody (Emfret, Würzburg, Germany, Clone No. JON/A, 1:20), in the presence or absence of thrombin agonist (0.1 U/mL, Chrono-log Corporation, Haverton, PA, USA). The assay was halted at 10 min by diluting the blood 1:5 with RT 1% PFA in Tyrode’s Buffer. Platelets were distinguished from white blood cells (WBCs) and debris based on size discrimination using forward scatter (FSC) and side scatter (SSC) and were defined as CD41+ singlets. Subsequently, 50 K platelets were captured and assessed for P-selectin and activated integrin αIIBβ3 expression. The specific monoclonal antibody (mAb) binding was quantified as the percentage of positive cells in the target gate.

Assessment of platelet-leukocyte aggregates: Immediately after anticoagulation with ACD, whole blood was diluted at 1:10 in M199 Media supplemented with 100 U/mL heparin. To characterize these aggregates, 300 μL of the diluted, citrated blood was dispensed into 2.0 mL tubes along with anti-mouse CD41-BV421 antibody (BioLegend, San Diego, CA, USA, Clone No. MWReg30; 1:150) and CD45-APC (BioLegend, San Diego, CA, USA, Clone No. 30-F11, 1:150). The blood samples were then stained for 15 min at 37 °C in darkness. After staining, the assay was terminated using 1.5 mL of BD FACS lysis buffer and fixed for 15 min in the dark at room temperature (RT). Subsequently, the samples were centrifuged at 800× *g* for 5 min, the supernatant was removed, and the fixed cells were resuspended in 600 μL of BD FACS lysis buffer. Platelet-leukocyte aggregates were classified by capturing 5 K CD45+ cells as a gating control, with platelets identified among all cells as CD41+. These aggregates were characterized as CD41+, CD45+. The specific monoclonal antibody (mAb) binding was quantified as the percentage of positive cells within the target gate.

Evaluation of lung platelets: Lung sections (5 microns) were immunostained by CD41 following established protocols [31,39]. Immunohistochemical staining and quantification were conducted on lung sections using anti-mouse CD41 antibody (Genetex, Irvine, CA, USA, GTX113758, 1:200), Dako EnVision + Dual Link System with anti-rabbit HRP DAB+ detection (Agilent, Carpinteria, CA, USA), and a light green counterstain (Statlab Medical Products, McKinney, TX, USA). The presence of distal lung platelets was evaluated by quantifying CD41+ pixels per high-powered field (×20). Lung regions containing significant vessels or airways were excluded from analysis, with 10 fields selected per mouse. CD41-positive pixels across entire lung sections were quantified using a random, unbiased approach employing ImageScope, version 12.4.3.500 (Leica Biosystems Imaging, Inc., Deer Park, TX, USA) pixel quantification software, following previously described methods [40].

ELISA: Platelet-poor plasma (PPP) and mouse lung samples were obtained following the previously described methods and stored at −80 °C until analysis. PF4 levels were quantified in both platelet-poor plasma (diluted 1:100) and 30 μg lung homogenates (diluted 1:200) using the mouse PF4 ELISA kit (Abcam, Cambridge, MA, USA)

Western blot: For lung homogenates, 30 µg of protein per well was loaded and separated by gel electrophoresis using Criterion XT 4–12% Bis-Tris Precast Gel (BioRad, Boulder, CO, USA) with XT MES running buffer (Bio-Rad). For plasma samples, 4 µL were loaded per well. Proteins were subsequently transferred to 0.2 µm polyvinylidene fluoride membranes (Bio-Rad) using a Trans-Blot Turbo rapid transfer system (Bio-Rad). Membranes were activated in methanol and blocked with 5% nonfat dry milk in Tris-buffered saline containing 0.05% Tween 20 (TBST) for 1 h. Following this, membranes were incubated with primary antibodies: EC-SOD mouse monoclonal antibody (sc 376948, Santa Cruz, Dallas, TX, USA) at 1:1000 in 5% milk in TBST at 4 °C overnight, and vinculin rabbit monoclonal (Cell Signaling, Danvers, MA, USA) at 1:10,000 in 5% milk in TBST at RT for 1 h. Subsequently, the appropriate horseradish peroxidase-conjugated anti-mouse or anti-rabbit secondary antibody (Santa Cruz) was applied at 1:10,000 in TBST for 1 h at RT. Detection was achieved using SuperSignal Femto Chemiluminescent substrates (Thermofisher, Waltham, MA, USA). Bands were quantified through densitometry using Image Laboratory Software (Bio-Rad) and normalized to the WT normoxia groups.

SOD activity assay: SOD activity was measured using a commercially available SOD assay kit (Dojindo Molecular Technologies, Mashiki, Japan) according to manufacturer instructions. In brief, this kit uses a water-soluble tetrazolium salt to produce a water-soluble formazan dye upon reduction with a superoxide anion, which is detectable via colorimetry. The rate of this reduction is linearly correlated to SOD concentration between 0.1 and 10 U/mL of SOD. The SOD activity, measured in units per ml of plasma, is then calculated based on a standard curve which is generated with xanthine oxidase and superoxide dismutase. 

Ex vivo SOD administration: Platelets from normoxic wildtype mice were isolated as above. To evaluate the effect of SOD on platelet activation, SOD isolated from bovine erythrocytes (Sigma-Aldrich) was added to the platelets at a concentration of 300 U SOD/mL platelets and incubated at RT for 3 min. Subsequently, platelets were then incubated for 10 min at RT in the dark, along with anti-mouse CD41-BV421 antibody (BioLegend, San Diego, CA, USA, Clone No. MWReg30; 1:50), P-selectin-APC (Thermofisher, Waltham, MA, USA, Clone No. Psel.KO2.3; 1:25), and activated integrin αIIBβ3-PE antibody (Emfret, Würzburg, Germany, Clone No. JON/A, 1:20), in the presence or absence of different activation agonist thrombin (0.1 U/mL, Chrono-log Corporation, Haverton, PA, USA), adenosine diphosphate (ADP) (5 uM, Chrono-log Corporation, Haverton, PA, USA) and Convulxin (350 ng/mL, Cayman Chemical, Ann Arbor, MI, USA), then evaluated the platelet activation by flow cytometry as described above.

Statistics: The data were analyzed using Prism 10 software (GraphPad; La Jolla, CA, USA). For all experiments, 6–10 male and female mice per group were used to provide a power of 80% to detect any effect size. For in vitro studies, we performed technical replicates (≥6) to ensure reproducibility and repeated experiments on at least two days. For experiments where two groups were assessed for significance, a student’s t-test was used. For experiments where three or more groups were assessed, either 1-way- or 2-way ANOVA with Tukey’s post hoc analysis was performed.

## 3. Results

**Figure 1:**
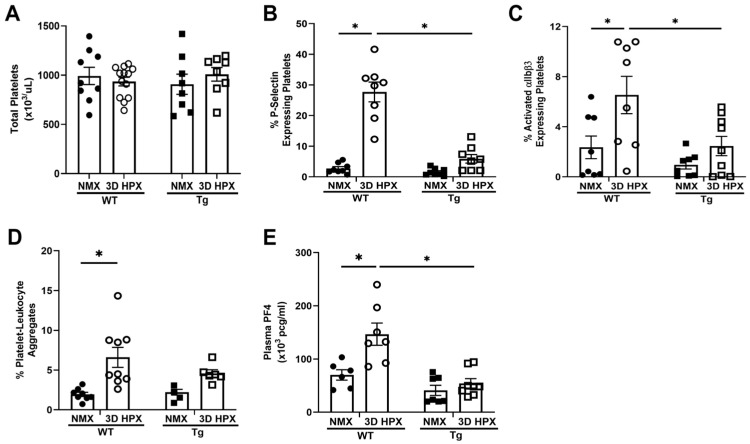
Platelet counts are similar across wildtype and EC-SOD overexpressing mice in normoxia and hypoxia, but platelet activation is attenuated by EC-SOD overexpression. (**A**) Using the Heska HT5 hematologic analyzer, total platelet counts were obtained from blood samples acquired from WT and lung EC-SOD overexpressing mice in both normoxia and hypoxia. N = 8–13. (**B**) P-selectin (Thermofisher) and (**C**) αIIBβ3 (Emfret) expression were assessed by flow cytometry in freshly isolated platelets from wildtype and lung EC-SOD overexpressing mice. N = 8, * *p* < 0.05 by two-way ANOVA. (**D**) Platelet-leukocyte aggregation was measured by co-expression of CD41-BV421 (BioLegend) and CD45-APC (BioLegend) antibodies. N = 4–9, * *p* < 0.05 by two-way ANOVA. (**E**) ELISA measuring PF4 (Abcam), a marker of platelet chemokine release, in platelet-poor plasma. N = 6–9, * *p* < 0.05 by two-way ANOVA. WT = Wildtype, Tg+ = mice overexpressing lung EC-SOD, NMX = normoxia, HPX = hypoxia, PF4 = platelet factor 4.

Hypoxia-induced platelet activation is prevented in lung EC-SOD overexpressing (Tg) mice. We previously demonstrated that lung EC-SOD overexpression protects against chronic hypoxic PH [21], though the specific mechanisms responsible for protection are unknown. Emerging data supports a key role for platelets in the pathogenesis of PH [41,42,43], and multiple molecules responsible for platelet activation are redox-sensitive [32,33,34]. Therefore, we tested whether EC-SOD Tg mice were protected against hypoxia-induced platelet activation. We observed no differences in blood total platelet count between WT and Tg mice at baseline or following 3 days of hypoxia (Figure 1A). Moreover, no differences across strains were observed in other hematologic indices, including MPV or Hgb at baseline or with hypoxia (Supplemental Figure S1). We found that platelet activation increased in hypoxic WT mice at day 3. This was demonstrated by increased activation of the integrin αIIBβ3 (Figure 1C) and increased release of the platelet alpha-granule stored proteins P-selectin (Figure 1B) and plasma PF4 (Figure 1E). Additionally, platelet-leukocyte aggregate formation (Figure 1D), a sensitive measure of in-vivo platelet activation, also increased in hypoxic WT mice at day 3. In contrast, in lung EC-SOD overexpressing Tg mice, hypoxia-induced platelet activation was prevented; hypoxia did not increase the expression of activated integrin αIIBβ3 (Figure 1C), P-selectin (Figure 1B), or plasma PF4 (Figure 1E). Similarly, platelet-leukocyte aggregation did not increase in Tg mice at day 3 of hypoxia (Figure 1D). 

**Figure 2:**
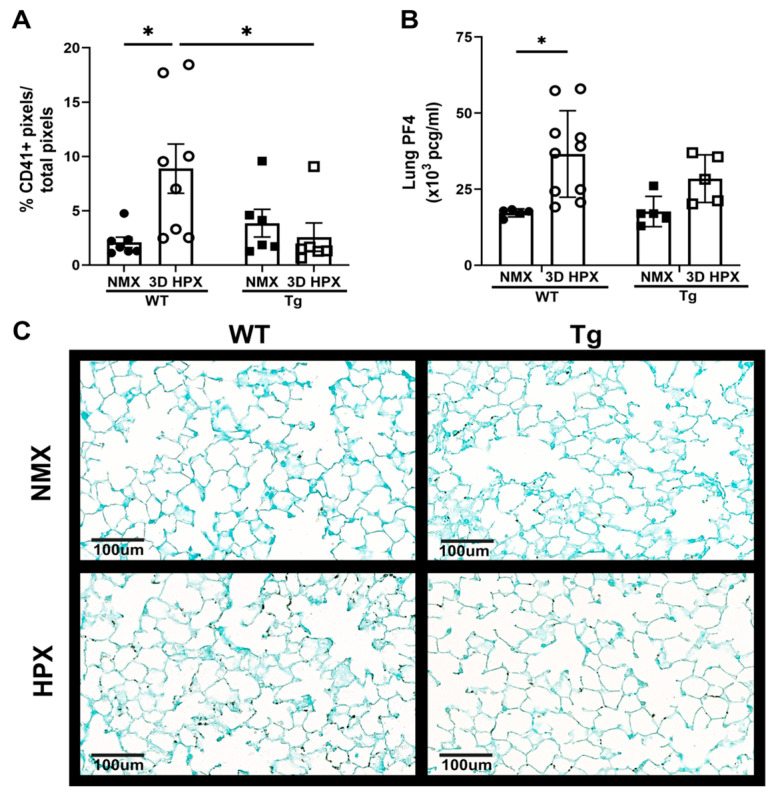
Hypoxia leads to increases in platelet infiltration and lung PF4 levels, which is prevented by EC-SOD overexpression. (**A**) Percentage of positive platelet marker CD41 (BioLegend) pixels in the lungs of wildtype mice and mice overexpressing lung EC-SOD in normoxia and hypoxia. N = 6–8, * *p* < 0.05 by two-way ANOVA. (**B**) Lung PF4 ELISA showing the concentration of PF4 in the lungs of wildtype mice and mice overexpressing lung EC-SOD exposed to normoxia or hypoxia. N = 5–10, * *p* < 0.05 by two-way ANOVA. (**C**) Representative histology (20x) images of CD41 (BioLegend) lung immunohistochemistry of the lungs of wildtype mice and mice overexpressing lung EC-SOD in normoxia and hypoxia. WT = Wildtype, Tg+ = mice overexpressing lung EC-SOD, NMX = normoxia, HPX = hypoxia, PF4 = platelet factor 4.

Hypoxia-induced accumulation of lung platelets and increased lung PF4 is prevented in mice overexpressing lung EC-SOD (Tg). We have previously demonstrated in WT mice that exposure to short-term hypoxia increases distal lung platelet accumulation and lung expression of the major platelet alpha-granule stored protein, PF4 [31]. To measure the effects of increased lung EC-SOD on lung platelet accumulation, we stained lung sections for the platelet-specific marker CD41+ and quantified platelet accumulation. Additionally, we performed ELISA on whole lung homogenates to quantify PF4 expression. Consistent with prior studies, lung platelets (Figure 2A,C), and lung PF4 (Figure 2B) increased following 3 days of hypoxia. In contrast, hypoxia-induced platelet accumulation (Figure 2A,C) was prevented in EC-SOD Tg mice. Similarly, levels of the platelet alpha-granule stored cytokine PF4 did not increase in the lungs of hypoxic Tg mice (Figure 2B). 

**Figure 3:**
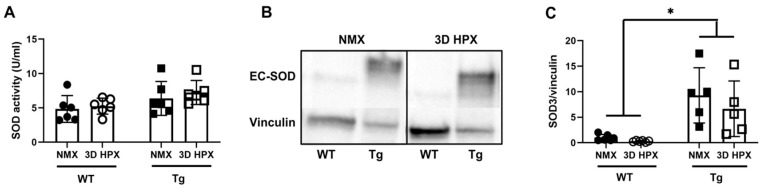
Plasma SOD activity was similar between mice overexpressing lung EC-SOD (Tg) mice and wildtype mice. (**A**) Plasma SOD activity in normoxic and 3-day hypoxic WT and Tg mice using a colorimetric assay (Dojindo). N = 6. (**B**) Representative western blot of lung EC-SOD expression along with housekeeping protein, Vinculin in WT mice and mice with lung overexpression of EC-SOD. (**C**) Densitometry of lung EC-SOD expression. N = 6, * *p* < 0.05 by two-way ANOVA. WT = Wildtype, Tg = mice overexpressing lung EC-SOD, NMX = normoxia, HPX = hypoxia.

Plasma SOD activity was similar between mice overexpressing lung EC-SOD (Tg) mice and wildtype mice. Given that platelet activation was tested in the circulation, we examined if lung overexpression of EC-SOD resulted in elevated plasma extracellular SOD activity. Plasma extracellular SOD activity was similar in WT and Tg mice at baseline and following 3 days of hypoxia (Figure 3A). Consistent with prior reports [21], total lung EC-SOD protein expression (endogenous mouse EC-SOD and human transgene EC-SOD) was higher at baseline in the lungs of EC-SOD overexpressing mice compared to WT controls and remained stable in each strain after exposure to 3 days of hypoxia (Figure 3B,C).

**Figure 4:**
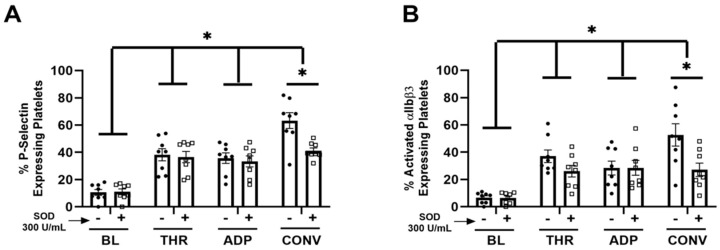
Ex vivo superoxide dismutase administration prevents convulxin-induced platelet activation. Freshly isolated platelets from wildtype mice were exposed to platelet agonists thrombin (0.1 U/mL, Chrono-log Corporation), adenosine diphosphate (ADP) (5 uM, Chrono-log Corporation) and Convulxin (350 ng/mL, Cayman Chemical), in the presence or absence of superoxide dismutase (SOD, 300 U/mL, Sigma Aldrich). Platelet activation was measured by expression (**A**) P-selectin and (**B**) activated αIIbβ3 via flow cytometry. N = 8, * *p* < 0.05. SOD = superoxide dismutase, BL = baseline, THR = thrombin, ADP = adenosine diphosphate, CONV = Convulxin.

Ex Vivo SOD treatment attenuated convulxin-induced platelet activation. To investigate the direct effect of extracellular SOD on platelet activation, freshly isolated platelets were pre-treated with SOD before stimulation with the platelet agonists thrombin, Convulxin, or ADP. Ex vivo SOD treatment did not affect thrombin or ADP-mediated platelet activation. However, ex vivo SOD treatment attenuated convulxin-induced platelet activation, demonstrated by attenuated expression of both platelet P-selectin (Figure 4A) and activated αIIbβ3 (Figure 4B). 

## 4. Discussion

We previously demonstrated that overexpression of lung EC-SOD protects against chronic hypoxic pulmonary hypertension [31]. Based on emerging data related to the redox control of platelet activation and the role of platelets in chronic hypoxic PH [25,32,34,41,44], we tested the hypothesis that overexpression of lung EC-SOD prevents hypoxia-induced platelet activation and accumulation of lung platelets. We now report that lung EC-SOD overexpression attenuates hypoxia-induced platelet activation in the circulation and platelet accumulation in the lung. Furthermore, we observed that exogenous extracellular SOD specifically blunted platelet activation due to Convulxin but not thrombin or ADP, suggesting modulation of GPVI-specific redox-sensitive targets. This study provides new insight into the role of lung EC-SOD on platelet activation, which has significant clinical implications for PH and other chronic hypoxic lung diseases.

This study presents two key findings. First, the overexpression of lung EC-SOD prevents platelet activation and accumulation of platelets in the lung in response to hypoxia. Our findings suggest that prevention of platelet activation may be one of the ways in which EC-SOD protects against the development of hypoxia-induced pulmonary vascular remodeling and PH. It is noteworthy that our studies were conducted in Colorado at 5280 ft elevation. The control mice in this study were maintained in Denver ambient air in contrast to other labs with ambient air at sea level or the use of hyperbaric chambers to simulate sea level. This choice of control atmospheric conditions was based on prior studies in our laboratory that demonstrated that chronic hypoxia resulted in a consistent and measurable phenotype, as well as a recent study that evaluated pulmonary hemodynamics in mice maintained in hyperbaric sea level chambers vs. mild hypoxia (Denver altitude) and did not observe differences in wild type mice under these two conditions [45]. Furthermore, platelet activation in mice bred at Denver ambient air but placed in hyperbaric sea level chambers had similar baseline platelet activation, measured by P-selectin expression, comparable to the control mice maintained in Denver ambient air in this study [31]. Thrombotic lesions are a common pathological finding in PH [46,47,48], and we recently demonstrated that platelets, a key component of thrombus formation, are increased in the lungs of patients with PH. Platelets are activated, and circulating platelet-derived proteins are increased in patients with PH [31] and recent studies reveal a strong correlation between platelet activation and the severity of PH [42,43,49]. Additionally, platelets from patients with PAH have high glycolytic rates, which correlate with hemodynamic alterations [50]. The effects of superoxide dismutase on platelet activation and recruitment have been previously studied. Administration of SOD abolishes collagen-mediated platelet activation and decreases platelet recruitment to a preformed thrombus after collagen stimulation [51]. Aged mice with platelet-specific SOD2 knockout have enhanced susceptibility to carotid and pulmonary artery thrombosis compared to WT mice and exhibit increased platelet-dependent thrombin generation [52]. Our findings underscore the multifaceted role of platelet activation in the pathophysiology of PH and highlight potential prognostic and therapeutic targets to monitor and mitigate disease progression.

We were interested in the compartmentalization of redox-sensitive processes and initially speculated that increased lung EC-SOD expression would elevate circulating SOD activity levels, however, we found no difference in plasma SOD activity between Tg and WT mice. Notably, lung EC-SOD overexpression prevented circulating platelet activation, despite unchanged plasma SOD activity. This raises the possibility that local EC-SOD is elevated in the pulmonary circulation, but this difference is not detected in the systemic circulation. Another possibility is that vascular EC-SOD content mitigates platelet activation via its regulation of resident lung cells such as endothelial cells through endothelial cell-platelet interactions. These are essential areas of future investigations. To begin to address this question, we tested the impact of exogenous SOD on platelet activation ex vivo by several major platelet activation agonists.

Our second major finding is that SOD targets specific pathways that result in platelet activation. Interestingly, we found that SOD attenuated Convulxin but not thrombin or ADP-mediated platelet activation. These platelet agonists work via different mechanisms. Convulxin activates the collagen receptor glycoprotein VI (GPVI) on platelets, leading to a signaling cascade that activates Syk, a tyrosine kinase, resulting in a cascade of reactions leading to thromboxane production, granule release, and integrin activation [53,54]. In contrast, soluble agonists such as thrombin and ADP trigger platelet activation through G-protein coupled receptors (GPCRs). Thrombin activates platelets via protease-activated receptors (PARs) on the platelet surface [28]. ADP is released from damaged endothelial cells and activated platelets and acts on P2Y1 and P2Y12 receptors [28]. Both thrombin and ADP increase cytosolic calcium-activating signaling pathways, resulting in granule release and integrin activation [55,56]. Given the potency of thrombin and ADP as platelet activators, it is possible that the effects of SOD were insufficient to overcome the activation due to the relatively high doses of thrombin and ADP that were used in our experiments. Alternatively, Convulxin may activate specific redox-sensitive molecules that are regulated by the extracellular redox environment and differ from thrombin and ADP. Published studies have implicated several interesting redox-sensitive targets in the GPVI activation pathway. First of all, the regulation of glycoproteins (GP), crucial for platelet function, is redox-regulated [57,58]. One of the mechanisms that regulates GP is proteolysis which is controlled by metalloproteinases. Increased proteolysis results in reductions in the density and reactivity of GP receptors which is important in platelet activation [57]. Studies have shown that A Disintegrin and Metalloproteinase domain-containing protein 10 (ADAM10) mediates GPVI shedding and that ADAM10 activity can be regulated via the oxidation of cysteine residues [59,60,61,62]. In addition, ROS have been shown to affect downstream targets of GPVI. For example, increased ROS can stimulate GPVI-induced Syk activation, representing a key redox-sensitive component of this platelet activation pathway that could be targeted by antioxidant strategies [62]. Lastly, it is worth noting that the differences in platelet activation could be due to changes in nitric oxide, which has a described role in thrombosis and platelet activation [63,64,65]. Superoxide dismutase catalyzes the dismutation of superoxide to hydrogen peroxide which also preserves NO activity [66,67]. These studies provide a strong rationale for future studies to examine the specific redox-regulated pathways responsible for platelet activation that can be regulated by the extracellular redox environment via EC-SOD.

## 5. Conclusions

Our study provides significant insight into the mechanisms by which lung EC-SOD protects against the development of hypoxic pulmonary hypertension. Our data demonstrate that increased EC-SOD prevents platelet activation and downstream inflammatory changes in both the plasma and the lung, processes that have been previously implicated in hypoxic pulmonary hypertension. Further studies should seek to understand the molecular mechanism of the redox-sensitive platelet activation pathways, as this would provide deeper insight and fully elucidate the potential of EC-SOD as a therapeutic agent for pulmonary hypertension.

## Data Availability

The original contributions presented in the study are included in the article/Supplementary Materials, further inquiries can be directed to the corresponding author/s.

## Supplementary Materials

The following supporting information can be downloaded at: https://www.mdpi.com/article/10.3390/antiox13080975/s1.
