# Supplementary material for: Lung EC-SOD Overexpression Prevents Hypoxia-Induced Platelet Activation and Lung Platelet Accumulation
Source: Antioxidants (Basel). 2024 Aug 10;13(8):975. doi: 10.3390/antiox13080975 (PMC11351248; doi:10.3390/antiox13080975)
Supplement: Supplementary file 1 [file antioxidants-13-00975-s001.zip › antioxidants-3112778-supplementary.pdf]

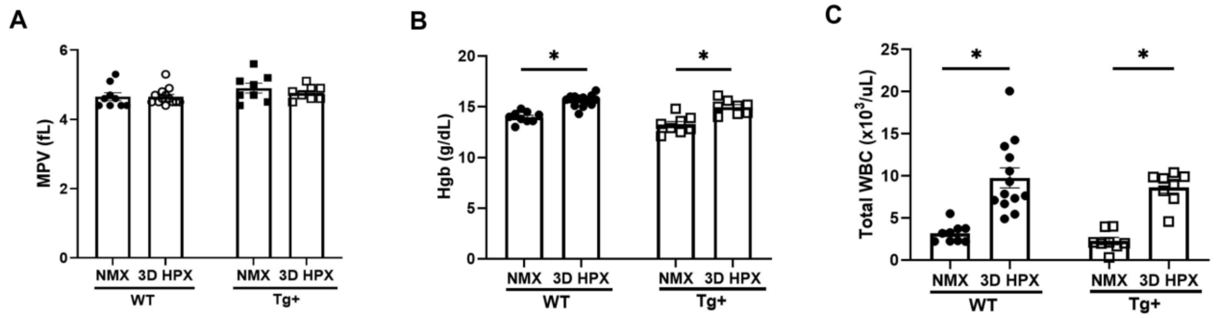

Supplemental Figure S1. Hypoxia increases hemoglobin and white blood cell count in both mouse strains. Blood was isolated from wildtype and Tg mice exposed to normoxia and hypoxia. Using the Heska HT5 hematologic analyzer, **A)** mean platelet volume, **B)** hemoglobin, and **C)** total white cell count were measured. N = 8–13, *$p < 0.05$. WT = Wildtype, Tg+ = mice overexpressing lung EC-SOD, NMX = normoxia, HPX = hypoxia, MPV = mean platelet volume, Hgb = hemoglobin, WBC = white blood cell.
